# Human exposure to soil contaminants in subarctic Ontario, Canada

**DOI:** 10.3402/ijch.v74.27357

**Published:** 2015-05-28

**Authors:** Ellen Stephanie Reyes, Eric Nicholas Liberda, Leonard James S. Tsuji

**Affiliations:** 1 School of Occupational and Public Health, Ryerson University, Toronto, Ontario, Canada; 2 School of Public Health and Health Systems, University of Waterloo, Waterloo, Ontario, Canada; 3 Health Studies and Department of Physical and Environmental Sciences, University of Toronto Scarborough, Toronto, Ontario, Canada

**Keywords:** DDT, soil, soil ingestion, risk assessment, Aboriginal health

## Abstract

**Background:**

Chemical contaminants in the Canadian subarctic present a health risk with exposures primarily occurring via the food consumption.

**Objective:**

Characterization of soil contaminants is needed in northern Canada due to increased gardening and agricultural food security initiatives and the presence of known point sources of pollution.

**Design:**

A field study was conducted in the western James Bay Region of Ontario, Canada, to examine the concentrations of polychlorinated biphenyls, dichlorodiphenyltrichloroethane and its metabolites (ΣDDT), other organochlorines, and metals/metalloids in potentially contaminated agriculture sites.

**Methods:**

Exposure pathways were assessed by comparing the estimated daily intake to acceptable daily intake values. Ninety soil samples were collected at random (grid sampling) from 3 plots (A, B, and C) in Fort Albany (on the mainland), subarctic Ontario, Canada. The contaminated-soil samples were analysed by gas chromatography with an electron capture detector or inductively coupled plasma mass spectrometer.

**Results:**

The range of ΣDDT in 90 soil samples was below the limit of detection to 4.19 mg/kg. From the 3 soil plots analysed, Plot A had the highest ΣDDT mean concentration of 1.12 mg/kg, followed by Plot B and Plot C which had 0.09 and 0.01 mg/kg, respectively. Concentrations of other organic contaminants and metals in the soil samples were below the limit of detection or found in low concentrations in all plots and did not present a human health risk.

**Conclusion:**

Exposure analyses showed that the human risk was below regulatory thresholds. However, the ΣDDT concentration in Plot A exceeded soil guidelines set out by the Canadian Council of Ministers of the Environment of 0.7 mg/kg, and thus the land should not be used for agricultural or recreational purposes. Both Plots B and C were below threshold limits, and this land can be used for agricultural purposes.

Food security exists “when all people, at all times, have physical and economic access to sufficient, safe and nutritious food to meet their dietary needs and food preferences for an active and healthy life” (1). In Canada, Aboriginal people (First Nations, Inuit, and Metis) are disproportionately food insecure compared to the Canadian general population – this is especially true for northern Aboriginal people – where up to 70% of the households in some areas were found to be food insecure (2,3). Indeed, remote Aboriginal people face unique food security issues related to the high cost of market food, as this type of food is typically flown in (4,5), and the high cost associated with hunting and fishing, such as cost of fuel to travel to hunting sites and financial expenses of owning hunting equipment (6,7). Thus, it is not surprising that food localization projects have been planned and initiated in northern Canada with respect to gardening, at both the small (home gardens and small greenhouses) and medium (community gardens and large greenhouses) scales (3,8,9). However, the soils used in these gardening initiatives have not been tested for contaminant levels, which is important because of known point sources in northern Canada for organochlorines from old radar lines (10–13) and metals from mines (14,15), as well as persistent organic pollutants (POPs) travelling long distances via atmospheric transport from industrial countries and deposited into the soil (16).

Importantly, during the Cold War in the 1950s the Mid-Canada Radar Line (MCRL) in subarctic Canada was built by the Government of Canada during the 1950s, in response to the threat of a nuclear attack from the Soviet Union (13,17). The MCRL was deemed redundant in the mid-1960s by the Canadian military and was decommissioned (13,17). However, since the radar-line stations were typically not properly decommissioned at the time of closure, many MCRL sites have become point sources of environmental contamination (e.g. polychlorinated biphenyls, PCBs; dichlorodiphenyltrichloroethane, DDT) (12,13). Site 050 (located on Anderson Island in close proximity to the community of Fort Albany First Nation, Ontario, Canada) was the first MCRL site to be remediated (12). This abandoned radar-line site received remediation priority because of elevated levels of PCBs in soil (21,000 ppm) and vascular plants (up to 550 ppm), with >50 ppm considered hazardous waste in Canada (12,17). Potential sources of DDT exposure also include DDT-contaminated soil surrounding MCRL buildings and long-range atmospheric transport from industrial sites, to the extent where remediation was required (16,18). The people of Fort Albany historically worked, lived, and partook in traditional activities (e.g. harvesting plants, berries, fish, and small game) on and around Anderson Island (11). Prior to remediation, there was potential for human exposure and uptake of PCBs and DDT from Site 050 (11).

During operation and when MCRL site 050 was abandoned, materials and equipment were moved off site and buried around the community of Fort Albany (11). Another potential source of soil contaminants in Fort Albany may be associated with the historical (into the 1970s) agricultural use of lands on the mainland, by the Roman Catholic Mission as 2 community members recount how they threw some “some powdery stuff” over the fields to control the pests during their time in residential school (8, p.6). The old Roman Catholic Mission agricultural fields and surrounding area were being considered for a new agroforestry initiative; agroforestry is a more sustainable land-use system than conventional agriculture, as it uses woody perennials with crops to optimize beneficial biological interactions (8,9).

Since organochlorine contaminants are highly lipophilic and resist biodegradation in the environment, they tend to bioaccumulate in biota and biomagnify up the marine food chain (19). Consequently, Arctic populations are exposed to greater concentrations because they are at the highest trophic level of the food chain and have more of a reliance on a subsistence diet (19,20). Soil contamination was a relevant issue from both direct exposure (e.g. ingestion and dermal contact) and indirect exposure (e.g. water ingestion, ingestion of vegetation, and exposure from the food chain) perspectives. Three potential plots of land were considered for the agroforestry initiative (8,9). In the present study, soil contamination was assessed in the 3 plots with respect to soil contaminant concentrations to inform the siting of the agroforestry initiative.

## Methods

### Description of study site

**Fig. 1:**
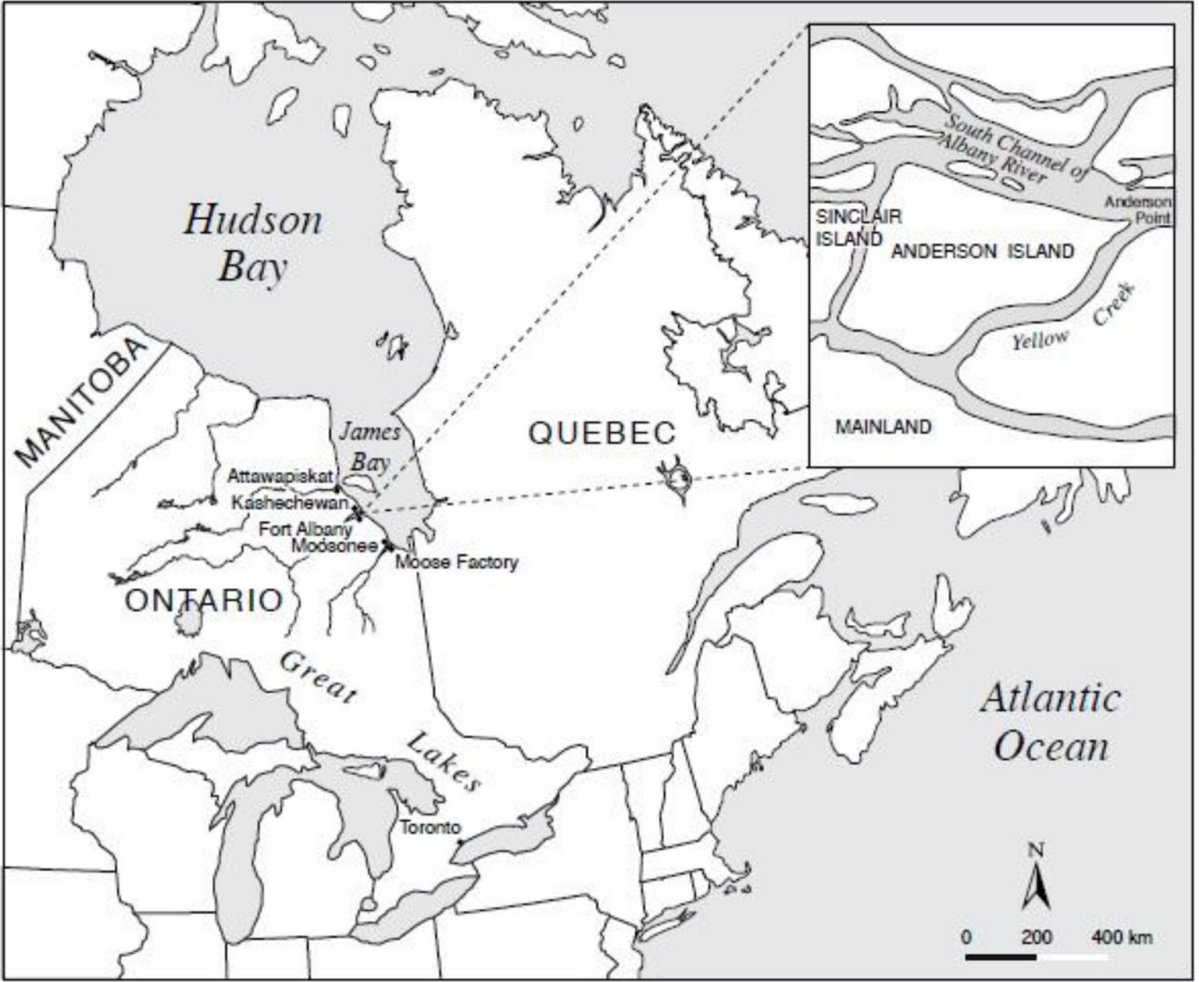
Location of the study site Fort Albany First Nations in Ontario, Canada.

Fort Albany First Nation is located on the western shore of the James Bay region (52°15′N, 81°35′W) of Ontario, Canada (21). Approximately 850 people live in the community (21). Figure 1 shows that the community is situated on Sinclair Island, but First Nation members also live on the Mainland and Anderson Island (21). MCRL Site 050 was located on Anderson Island. As Fort Albany is a remote community, accessibility is limited with barges during late spring to early fall, ice/snow during the winter, and year-round access by aircraft (13).

### Field sample collection

Ninety soil samples were collected at random (grid sampling) from 3 plots (A, B, and C) in Fort Albany (on the mainland), subarctic Ontario, Canada. The plot areas were 10×10 feet and the soil samples were taken at root level due to concerns with potential plant uptake of contaminants. Six inch sample cores were collected and weighed before being refrigerated and shipped in Ziploc^®^ bags for analysis. All soil samples were prepared and analysed for PCBs, DDT and its metabolites, other organochlorines, and metals/metalloids at the Analytical Services Unit, Queen's University, Kingston, Ontario.

### Sample preparation

#### Sample preparation for organochlorines

Samples were thoroughly homogenized before sampling for extraction and cleanup. Soil samples were subsampled for determination of wet/dry weight ratio. Accurately weighed 10 g of soil sample to which an aliquot of surrogate standard, dechlorobiphenyl, 40 g of sodium sulphate, and 20 g of Ottawa sand were added. Samples were extracted 3 times for 20 minutes with 50 mL of dichloromethane on an orbital shaker. The extract was then concentrated by rotoevaporation to approximately 1 mL, and 5 mL of hexane was added and again evaporated to 1 mL. This was repeated twice more, resulting in 1 mL of hexane solvent, which was then applied to a Florisil column for cleanup. The column was thoroughly rinsed with hexane and the eluent containing the organochlorines diluted to 10.0 mL. A 2-mL gas chromatography (GC) vial was then filled.

#### Sample preparation for metals/metalloids (except for Hg)

Samples were air-dried and ground to a fine powder with a mortar and pestles. Large stones were removed from the soil samples, as they would not be expected to contain any anthropogenic environmental contaminants. Accurately weighed 0.5 g of powdered soil sample was heated with 2 mL of nitric acid (HNO_3_) and 6 mL of hydrochloric acid (HCl) and reduced the volume to 1–2 mL. This solution was made up to 25 mL with deionized water and filtered through a Whatman No. 40 filter paper.

### Analysis and quality assurance

#### Analysis for organochlorines

Soil samples were analysed for total PCBs (Aroclor 1016, Aroclor 1221, Aroclor 1232, Aroclor 1242, Aroclor 1248, Aroclor 1254, and Aroclor 1260), total DDT and its metabolites [total DDT refers to the sum of all DDT-related compounds, ΣDDT, *p,p*′-DDT, *o,p*′-DDT, *p,p*′-DDE (dichlorodiphenyldichloroethylene), *o,p*′-DDE, *p,p*′-DDD (dichlorodiphenyldichloroethane), and *o,p*′-DDD],^1^ and other organochlorines [α-hexachlorocyclohexane (HCB), β-HCB, γ-HCB, δ-HCB, heptachlor, aldrin, heptachlor epoxide isomer B, endosulfan I, dieldrin, endrin, endosulfan II, endrin aldehyde, endosulfan sulphate, and methoxychlor]. Each sample was analysed using an Agilent 6890 gas chromatography equipped with a ^63^Ni electron capture detector (GC/ECD), a SPB^TM^-1 fused silica capillary column 90 m, 0.25 mm ID×0.25 µm film thickness). A fraction may be analysed by gas chromatography with a mass spectrophotometer as a detector (GC/MS) if interfering compounds were present. The GC/MS analysis used the following: an HP 5890 Series II Plus gas chromatograph equipped with an HP 5972 Mass selective detector and a PTETM-5 fused silica capillary column (30 m, 0.25 mm ID×0.25 mm film thickness).

#### Analysis and quality assurance for metals/metalloids (except for Hg)

A 30-element suite of metals/metalloids was analysed: Ag, Al, As, B, Ba, Be, Ca, Cd, Co, Cr, Cu, Fe, K, Mg, Mn, Mo, Na, Ni, P, Pb, S, Sb, Se, Sn, Sr, Ti, Tl, U, V, and Zn. Samples were analysed in batches of up to 36, which comprised up to 28 samples, 2 blanks, 4 duplicates, and 2 reference materials, Mess-3 and SS-2, from the National Research Council of Canada. The control limits for Mess-3 and SS-2 are in Supplementary Tables I and II. All samples were analysed by an inductively coupled plasma mass spectrometer.

**Table I:** Soil quality guidelines for DDT, PCBs, and some metals/metalloids (mg/kg)

	Land use			
	
Contaminant	Agricultural	Residential/parkland	Commercial	Industrial
Total DDT guideline	0.7	0.7	12	12
Total PCBs guideline	0.5	1.3	33	33
Inorganic As guideline	12	12	12	12
Ba guideline	750	500	2,000	2,000
Cd guideline	1.4	10	22	22
Total Cr guideline	64	64	87	87
Cu guideline	63	63	91	91
Ni guideline	50	50	50	50
Pb guideline	70	140	260	600
Se guideline	1	1	2.9	2.9
U guideline	23	23	33	300
V guideline	130	130	130	130
Zn guideline	200	200	360	360

“Canadian Soil Quality Guidelines for the Protection of Environmental and Human Health: Summary Table” by the Canadian Council of Ministers of the Environment (24). DDT, dichlorodiphenyltrichloroethane; PCBs, polychlorinated biphenyls.

**Table II:** Exposure factors for calculating the soil exposure analysis

	Values by age group		
	
Exposure factors	5–11 years	12–20 years	21+years
Soil ingestion rate (mg/day)	400	400	400
Total soil adherence (mg/day)	5,800	9,100	8,700
Body weight (kg)	27	57	70
Bioavailability factor (unitless)	1.0	1.0	1.0
Exposure duration per week (days)	5	5	5
Exposure duration per year (weeks)	52	52	52
Exposure duration in a lifetime (years)	8	16	30
Lifetime (years)	12	20	70

The default EF values come from “Investigating Human Exposure to Contaminants in the Environment: A Handbook for Exposure Calculations” by Health Canada (26). The recommended maximum estimate of soil ingestion rate of 400 mg/day is based on a study by Harper et al. (27).

#### Analysis and quality assurance for mercury

The Hg analysis was determined by cold vapour atomic absorption spectrophotometry. The instrument, a direct mercury analyser (DMA-80), allowed for measurement with little to no sample preparation as described in the U.S. Environmental Protection Agency (EPA) Method 7473 (23). Samples, which were weighed into quartz or nickel boats, enter the instrument's chamber where the sample is first dried and then thermally decomposed in a continuous flow of oxygen (O_2_). The combustion products are carried off in the O_2_ and are then further decomposed in a hot catalyst bed. The Hg vapours are trapped on a gold amalgamator tube and desorbed for spectrophotometric quantitation at 254 nm. Quality assurance/quality control procedures included method blanks and laboratory control samples throughout the entire sample preparation and analytical process (23).

### Soil quality guidelines

The Canadian Council of Ministers of the Environment (CCME) developed soil quality guidelines for total DDT, total PCBs, and some metals/metalloids for the protection of the environment and human health (24). Table I presents the soil quality guidelines depending on land usage. The key total DDT metabolites that are found in the northern environment are *p,p*′-DDT, *o,p*′-DDT, and *p,p*′-DDE. Since DDT is a persistent chemical that tends to bioaccumulate and biomagnify throughout the food chain, as well as contribute with atmospheric transport to the Arctic from industrial areas, the concept of land usage has to be taken into consideration, particularly with agricultural and residential/parkland (22). For the purposes of this study, comparisons are made between the soil quality data from the study site in Fort Albany with the guideline for total DDT, total PCBs, and metals/metalloids from the agricultural and residential/parkland usage.

### Soil exposure factors and calculations

Ideally, all pathways should be considered when estimating the daily intake of a chemical contaminant. However, only the exposure factors (EFs) for soil ingestion and soil dermal uptake were accounted for as these are the likely exposure routes as shown in Table II. The default factor values used in risk assessment analyses used to estimate exposure to pesticides frequently overestimate exposure but are the first preference for assessing the safety implications for the community (25). For instance, the exposure duration is 5 days a week for 52 weeks per year; however, the actual exposure duration is not as long. The default EFs were stratified according to age group to indicate how often the individual is exposed to the contaminant during a year and the number of years this pattern has been repeating (26).

Health Canada's recommendations for maximum estimate of soil ingestion intake of 35 mg/day for the 5–11 year age group, and 20 mg/day for the 12–20 year and 21+ year age groups were not used for this study (26). A soil ingestion study done by Harper et al. (27) recommended a maximum estimate of soil ingestion rate of approximately 400 mg/day because it is a conservative parameter used to evaluate health risks associated with the contaminated sites. This EF is based on Aboriginal practices that involve consuming traditional food sources that can become contaminated with soil particles, gardening, gathering, and preservation techniques that can increase the level of soil contact, and other additional environmental activities (e.g. outdoor recreation for children and cultural activities) (27,28). As a worst-case scenario, a bioavailability value of 1 (100%) was used in exposure estimation. The EF is calculated to estimate an average dose over the exposure period Health Canada (26) as provided in the following equation:

$$\text{EF}=\frac{\begin{array}{l} \text{exposure duration per week}\left(\text{day}/\text{week}\right)\\ \times \text{exposure duration per year}\left(\text{weeks}/\text{year}\right)\\ \times \text{exposure duration in a life time}\left(\text{years}\right) \end{array}}{\text{life time}\left(\text{years}\right)\times \text{365 days}/\text{year}}$$

The amount of total DDT absorbed into the body by soil ingestion (ED_s_) is estimated with the following equation (26):

$${\text{ED}}_{s}=\frac{C\times \text{IR}\times \text{EF}\times 10^{-6}}{\text{BW}}$$

where ED_*s*_=is the estimated dose through soil ingestion expressed as milligrams of contaminant eaten per kilogram of body weight per day (mg/kg/day); *C*=the concentration of the contaminant in the soil in milligrams per kilogram of soil (mg/kg); IR=the soil ingestion rate, the amount of soil an individual eats in a day in milligrams (mg/day); EF=the exposure factor, which indicates how often the individual has been exposed to the contaminant over a lifetime; and BW=the body weight, that is, the average body weight in kilograms based on an individual's age group (kg).

The amount of total DDT that is absorbed into the body through dermal contact with contaminated soil (ED_ss_) can be estimated with the following equation (26):

$${\text{ED}}_{ss}=\frac{C\times A\times \text{BF}\times \text{EF}\times 10^{-6}}{\text{BW}}$$

where ED_ss_=is the estimated dose through dermal contact with soil expressed as milligrams of the contaminant absorbed through the skin per kilogram of body weight per day (mg/kg/day); *C*=the concentration of the contaminant in the soil in milligrams per kilogram of soil (mg/kg); *A*=the total soil adherence, amount of soil that sticks to an individual expressed in milligrams per day; BF=the bioavailability factor, the percentage of the contaminant in the soil that is actually free to move out of the soil and through the skin (unitless); EF=the exposure factor, indicates how often the individual has been exposed to the contaminant over a lifetime; and BW=body weight, average body weight in kilograms based on an individual's age group (kg).

### Recommended estimated maximum intake values

Regulatory agencies developed guidelines and advisories regarding the usage of DDT, DDE, and DDD in the environment. The details of the recommended intake values applicable to DDT are summarized in Table III.

**Table III:** Regulatory guidelines of recommended estimated maximum intake values for DDT

Chemical	Regulatory agency	Toxicity value	Details about study and references
DDT	U.S. EPA	Acute oral MRL^a^: 0.0005 mg/kg/day	MRL of 0.0005 mg/kg/day based on a lowest observed adverse effect level (LOAEL) of 0.5 mg/kg/day for neurodevelopmental effects in mice. Applied uncertainty factor (UF) of 1,000 (10 for use of LOAEL, 10 for animal to human extrapolation, and 10 to account for intrahuman variation) (29)
DDT	U.S. EPA	Intermediate oral MRL: 0.0005 mg/kg/day	MRL of 0.0005 mg/kg/day based on a NOAEL of 0.05–0.09 mg/kg/day for liver effects in Osborne–Mendel rats administered technical DDT in the diet at the dosage of 0, 1, 5, 10, or 50 ppm for 15–27 weeks (29)
*p,p*′-DDT	Integrated Risk Information System (IRIS) developed by the U.S. EPA	RfD^b^: 0.0005 mg/kg/day	Critical effect: liver lesions with a 27-week rat feeding study. No observable effects limit (NOEL): 1 ppm diet. LOAEL: 5 ppm. Applied a UF of 100 and modifying factor (MF): 1 (30,31)
DDT	Health Canada	TDI^c^: 0.01 mg/kg/day	No observed adverse effect level (NOAEL) of 1 mg/kg of body weight per day from a 7-month developmental toxicity in rats. Applied UF of 100 (32,33)

a Minimal risk level (MRL): an estimate of daily human exposure to a hazardous substance that is likely to be without appreciable, non-carcinogenic health effects over a specified duration of exposure (34).

b Reference dose (RfD): an estimated daily oral exposure of a chemical to the human population (including sensitive groups) that is likely to be without an appreciable health risk over a lifetime (35).

c Tolerable daily intake (TDI): an estimated amount of a substance in food, drinking water, or air that can be ingested over a lifetime without deleterious, non-carcinogenic effects (36).

## Results

### Soil quality

The concentration ranges of total DDT found in the soil plots were distributed heterogeneously with values ranging from below the detection limit to 4.19 mg/kg. Table IV indicates that Plot A had the highest total mean DDT concentration of 1.12 mg/kg, followed by Plot B and Plot C, which were 0.09 and 0.01 mg/kg, respectively. The concentrations of the PCBs and other organochlorines were below the detection limit and hence we have not presented the data as these contaminants do not pose a health risk.

**Table IV:** Concentration of ΣDDT (mg/kg) from the 3 contaminated-soil plots

	Plot A				Plot B				Plot C			
			
	Mean	SD	Min	Max	Mean	SD	Min	Max	Mean	SD	Min	Max
Total DDT	1.12	1.66	0.09	4.19	0.09	0.04	0.04	0.19	0.01	0.01	0.00	0.03

The metal concentrations in the 3 soil plots are presented in Supplementary Table III. In comparison to the available Canadian soil quality guidelines from Table I, contamination in the plot sites by metal pollutants is not of concern. The concentration levels of the toxic metals are well below the soil quality guidelines, and thus meet the benchmark for safe usage of relevant land resources (24).

### Soil exposure analysis for DDT

**Fig. 2:**
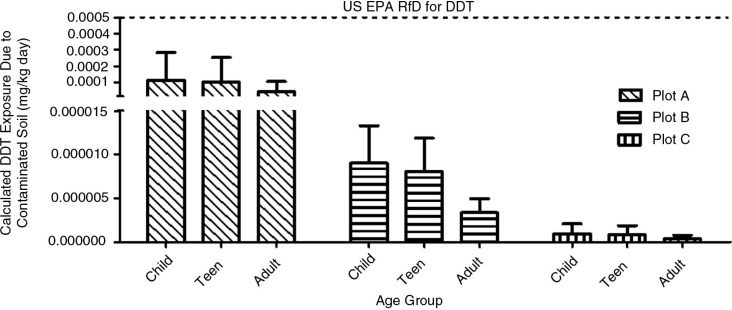
Calculated DDT exposure by direct ingestion compared to the U.S. EPA Reference Dose.

To assess the potential health risks due to the contamination of DDT compounds in the soil plots, exposure model calculations were applied. Figure 2 presents the exposure to total DDT by direct soil ingestion. The data clearly showed that Plot A had a much higher level of DDT compounds compared to B and C. In general, the level had the order: child>teen>adult. The mean exposure concentration±standard deviation of total DDT by soil ingestion was 4.33×10^−5^±5.04×10^−5^ mg/kg/day, with ranges not detectable to 9.68×10^−4^ mg/kg/day.

Table V shows that the estimated daily intake (EDI) of total DDT was averaged to be 4.35×10^−5^ mg/kg/day over a lifetime of 70 years (hazard index=0.00435). The EDI is tabulated by adding each possible combination of exposure pathway, and it is noted that the estimated dose is calculated separately for each age group. The results show that soil dermal uptake is the main exposure pathway to total DDT.

**Table V:** Estimated daily intake of ΣDDT averaged over a lifetime of 70 years

	Age (years)			
	
Pathway	5–11	12–20	21+	Daily exposure
Soil ingestion	1.15×10^−7^	3.75×10^−8^	1.78×10^−8^	1.71×10^−7^
Soil skin exposure	1.91×10^−5^	1.71×10^−5^	7.11×10^−6^	4.33×10^−5^
Total	1.92×10^−5^	1.71×10^−5^	7.13×10^−6^	4.35×10^−5^

All values expressed in mg/kg/day.

## Discussion

### Comparison of soil quality guidelines and recommended maximum intake values

There was a high degree of variability for total DDT between each soil plot. The total DDT levels of both Plots B and C (0.09 and 0.01 mg/kg, respectively) were orders of magnitude below the maximum threshold limits developed by CCME for agricultural and parkland/usage of 0.7 mg/kg (22). Plot A had a total DDT level of 1.12 mg/kg and this result was higher than Canada's soil guidelines. It is worth mentioning that DDT was prohibited and removed from major use in Ontario since 1972 but still persists in the natural environment (13,37). Since Plot A exceeded government guideline threshold limits, this plot should not be used for agricultural or recreational purposes.

Interestingly, the results from the present study indicate that if there were potential health concerns due to DDT exposure, this would mainly occur through dermal contact. This finding contrasts with other studies that suggest that direct ingestion is the most common route of exposure to DDT (16,38,39). However, these studies noted that food consumption is the main source of intake. Bard's (16) study focused on POP contamination from consuming fish and other marine mammals, and Dougherty et al. (38) and MacIntosh et al.'s (39) studies assessed the potential hazards of consuming various food products, such as milk, beef, and fish, as sources of exposure to DDT. Lastly, the results from the estimated exposure assessment to total DDT by direct soil ingestion were below regulatory guidelines set out by the U.S. EPA and Health Canada for all soil plots and age groups. Since the results from the soil exposure analysis were below the reference dose and tolerable daily intake, this indicates that the exposure level to DDT via the soil is not likely to pose any risk to human health, even using a bioavailability factor of 1 (100%).

### Comparison of soil samples from different locations

Table VI compares the results from this study to other contaminated-soil studies from different locations in Canada and globally. Sites B and C in the present study had ΣDDT soil levels similar to those reported for Prince Albert National Park, Saskatchewan, Canada (range: <LoD to 0.15 mg/kg), a non-agricultural location (40). Although Site A had ΣDDT soil levels above the guidelines for agricultural use in Canada, mean ΣDDT soil concentration was comparable to that reported for Canadian soil sampled at Point Pelee, Ontario (mean: 1.21 mg/kg) and Fraser Valley, British Columbia, Canada (mean: 4.06 mg/kg) or less than that found in agricultural regions of Ontario for the Niagara Peninsula (range: <LoD to 14.4 mg/kg) and Holland Marsh (mean: 19 mg/kg) (41–44). The increased application volume of DDT to agricultural areas in Ontario, especially in orchard soils and vegetable fields, during the 1940–1970s, has led to the relatively elevated ΣDDT soil levels reported (41,43).

**Table VI:** Mean ΣDDT levels compared with other Canadian and global soil sites

Country	Location	Land use	Mean ΣDDT (mg/kg)	References
Canada	Fort Albany, Ontario	Gardening/agricultural initiative	Plot A: 1.12 Plot B: 0.09 Plot C: 0.01	Present study
Canada	Saskatchewan	National park and rural agricultural sites	<LoD to 1.50×10^−1^^a^	Bailey et al. (40)
	Point Pelee, Ontario	Marsh and natural sand dunes; former agricultural, residential, and youth camp areas	1.21^b^ (1.86×10^−3^ to 316^a^)	Crowe and Smith (41)
	Fraser Valley, British Columbia	Agricultural soil	4.06	Finizio et al. (42)
	Niagara Peninsula, Ontario	Fruit orchard soils	<LoD to 14.4^a^	Harris et al. (43)
	Holland Marsh, Ontario	Historically treated agricultural soils	19	Kurt-Karakus et al. (44)
China	Tibet	Soil near mountainous and polar regions	<LoD to 2.83×10^−3^^a^	Fu et al. (45)
	Tianjin	Surface agricultural soils	5.60×10^−2^	Tao et al. (46)
	Haihe Plain	Surface soil as a re-emission source	6.36×10^−2^	Tao et al. (47)
	Beijing	Industrial soil site with future residential development	3.02 to 67.43	Yang et al. (48)
East Antarctic	Novolazarevskaya	Soil without vegetation and location used to deposit equipment and fuel	Plot 1: 2.43×10^−3^ Plot 2: 2.81×10^−3^ Plot 3: 3.18×10^−3^	Negoita et al. (49)
	MoLoDezhnaya	Fuel reservoirs	Plot 1: 9.13×10^−3^ Plot 2: 2.65×10^−2^ Plot 3: 1.22×10^−3^	
	Stornes Peninsula	Soil without vegetation	1.16×10^−3^	
	Druzhnaya	Soil without vegetation	1.10×10^−4^	
	Progress	Soil without vegetation	1.17×10^−3^	
	Mirny (Haswell Archipelago)	Natural reservation and frequented by birds and penguins	Plot 1: 6.28×10^−3^ Plot 2: 8.14×10^−3^	
India	District Dibrugarh	Agricultural fields, fallow and urban lands	7.57×10^−1^ (7.50×10^−2^ to 2.30^a^)	Mishra et al. (50)
	District Nagon	Agricultural fields, fallow and urban lands	9.03×10^−1^ (1.66×10^−1^ to 2.29^a^)	
	Agra	Agricultural, nursery, gardening, and landfill areas	1.01	Singh (51)
Poland	Katowice	Surface soil	1.10×10^−1^ (2.30×10^−2^ to 2.60×10^−1^^a^)	Falandysz et al. (52)
	Kraków	Surface soil	2.60×10^−1^ (4.30×10^−3^ to 2.40^a^)	

a Minimum and maximum values of ΣDDT.

b Geometric mean of ΣDDT.

In comparison to soils from southern Poland for the cities of Katowice (mean: 0.110 mg/kg) and Kraków (mean: 0.260 mg/kg), the contamination level of DDT found in Fort Albany is similar (52). The soil plots in India for the District Dibrugarh (mean: 0.757 mg/kg), District Nagaon (mean: 0.903 mg/kg), and Agra (mean: 1.01 mg/kg) have relatively elevated total DDT values compared to Fort Albany (50,51), which is not surprising, as these soil concentration values are for a country that continues to produce and use organochlorine pesticides such as DDT as a vector control agent (50).

Both China and East Antarctic have lower contamination levels of total DDT compared to Fort Albany with ranges from <LoD to 6.36×10^−2^ mg/kg (45–47, 49). However, the exception was an industrial soil site in Beijing, China, that had unevenly distributed total DDT concentrations of 3.02–67.43 mg/kg in different soil layers (48). This is of particular concern since this contaminated-soil site is currently a paint factory, but plans are developed for future restoration and residential development (48).

### Limitations

Due to the close proximity of Fort Albany First Nations to MCRL Site 050, our group had reason to suspect contamination of the soil. This was further compounded by the traditional ecological knowledge of the community elders who recall chemicals being applied to the land. We chose to focus on a variety of contaminants including DDT, PCBs, and metals; however, it is possible that other contaminants are present. Further, while there are other communities nearby, it is not known if their soils have similar contaminants. Future studies will investigate the soil concentrations of environmental contaminants in other communities to determine if a point-source exposure can be located and to safeguard human health.

## Conclusion

The soil used in the First Nation community of Fort Albany, Ontario, Canada, is primarily contaminated by DDT, but also by low concentrations of some metals/metalloids (e.g. As, Ba, Co, Cr, Cu, Ni, Pb, V, and Zn). The soil exposure and estimated analysis revealed no known health risks to humans, as the results were well below government thresholds recommended by the U.S. EPA and Health Canada, even though the ΣDDT concentration in the Plot A soil was above Canada's soil quality guidelines. Nonetheless, it is prudent for any agricultural initiative in northern Canada to first test the soil for contamination, as there are many sources of contaminants in the north other than long-range transport that may impact the quality of the food produced. The methods in the Fort Albany, Ontario study can also be used to measure contaminants in soil of communities located near known or suspected point sources of pollution, such as military sites (e.g. White Alice sites in Alaska) (53) and near extraction industries (e.g. Russia's European High North) (54) to address environmental health concerns across the Circumpolar North.

## Supplementary Material

Human exposure to soil contaminants in subarctic Ontario, Canada

## Footnotes

1 The typical percentages of each of the isomers is 77% *p*,*p*′-DDT, 15% *o*,*p*′-DDT, 4% *p*,*p*′-DDE, 0.1% *o*,*p*′-DDE, 0.3% *p*,*p*′-DDD, 0.1% *o*,*p*′-DDD, and a number of unidentified compounds (3.5%) (22). The typical percentages are added to highlight the importance of measuring each isomer separately.
