# Supplementary material for: Human exposure to soil contaminants in subarctic Ontario, Canada
Source: Int J Circumpolar Health. 2015 May 28;74:10.3402/ijch.v74.27357. doi: 10.3402/ijch.v74.27357 (PMC4449361; doi:10.3402/ijch.v74.27357)
Supplement: Human exposure to soil contaminants in subarctic Ontario, Canada [file IJCH-74-27357-s001.pdf]

SUPPLEMENTARY MATERIAL

Table 1. Metal Control and Detection Limits for Mess-3

Element	Wavelength	Reference Value	Mean	Warning Limits	Control Limits
Al	237.312	85900	22000	15000-28900	11600-32300
As	193.696/188.98	21.2	17.4	15.6-19.1	14.8-20.0
Ba	585.367	-	357	293-422	261-454
Ca	370.602	14700	13295	12000-14600	11300-15300
Co	228.615	14.4	12.1	10.6-13.6	9.8-14.3
Cr	267.716	105	41.0	30.3-52.0	24.6-57.5
Cu	324.754	33.9	30.8	27.8-33.7	26.4-35.2
Fe	260.709	43400	34800	31900-37800	30400-39200
K	766.491	(26000)	5490	3900-7090	3100-7890
Mg	383.829	(16000)	13100	12000-14300	11500-14800
Mn	294.921	324	306	274-337	258-353
Mo	202.032	2.8	2.0	<2.0-2.9	<2.0-3.4
Na	330.237	(16000)	10900	10000-11900	9540-12400
Ni	231.604	46.9	38.3	34.4-42.2	32.5-44.1
P	213.618	(1200)	1020	885-1150	818-1220
Pb	220.353	21.1	18.1	16.4-19.8	15.6-20.6
S	181.972	(1900)	1670	1490-1850	1400-1940
Sr	346.45	129	65.5	57.1-73.9	52.9-78.1
V	292.401	243	88.8	63.3-114	50.5-127
Zn	206.200	159	135	122-148	116-154

Warning limits are two standard deviations and control limits are three standard deviations. Reference values obtained from National Research Council Canada data sheet.

Table 2. Metal Control and Detection Limits for SS-2

Element	Wavelength	Reference	Mean	Warning Limits	Control Limits
Al	237.312	13265, 44853	17500	14700-20200	13300-21600
As	193.696/188.98	21.2	17.4	15.6-19.1	14.8-20.0
Ba	585.367	215, 650	241	208-277	187-295
Ca	370.602	112861,	121000	95100-147000	82000-160000
Cd	214.439	(2)	1.9	1.4-2.3	1.2-2.5
Co	228.615	12, 14	14.0	12.0-16.0	11.0-17.0
Cr	267.716	34, 58	43.1	36.3-49.8	32.9-53.2
Cu	324.754	191, 198	190	164-217	150-230
Fe	238.204	21046, 29070	26600	23200-30000	21500-31800
K	766.491	3418, 18119	4630	3790-5480	3370-5900
Mg	383.829	11065, 14225	12700	10900-14400	10100-15200
Mn	294.921	457, 577	545	479-612	445-645
Mo	202.032	(4)	2.6	<2.0-3.5	<2.0-4.0
Na	589.592	558, 12539	766	554-978	449-1080
Ni	231.604	54, 59	53.5	47.5-59.5	44.5-62.5
P	213.618	752, 814	682	534-924	437-1020
Pb	220.353	126, 148	116	101-130	93.7-138
S	181.972	(2193), (2254)	2179	1920-2440	1780-2570
Sn	189.927	(6)	2.6	<2.0-4.2	<2.0-5.0
Sr	346.445	214, 382	217	194-240	182-252
Ti	334.188	850, 2893	1340	857-1820	616-2060
V	292.401	34, 59	47.9	39.4-56.5	35.1-60.8
Zn	206.200	467, 509	454	400-507	373-534

Warning limits are two standard deviations and control limits are three standard deviations. Reference values obtained from National Research Council Canada data sheet.

Table 3. Metal Concentrations (ppm) in the Three Potential Agroforestry Plots

Element	Plot A				Plot B				Plot C			
	Mean	SD	Min	Max	Mean	SD	Min	Max	Mean	SD	Min	Max
Ag	0.0	0.0	0.0	0.0	0.0	0.0	0.0	0.0	0.0	0.0	0.0	0.0
Al	7171.1	537.4	6380.0	7970.0	6922.9	667.8	5430.0	8950.0	6975.6	411.5	6490.0	7770.0
As	2.1	0.2	1.9	2.3	1.7	0.3	1.4	2.5	2.1	0.2	1.7	2.4
B	19.7	7.6	0.0	25.2	24.4	2.0	20.9	27.7	24.5	1.5	22.3	27.8
Ba	41.9	3.3	38.4	49.7	40.9	2.0	36.3	44.7	40.6	3.6	36.9	49.4
Be	0.0	0.0	0.0	0.0	0.0	0.0	0.0	0.0	0.0	0.0	0.0	0.0
Ca	51722.2	3683.2	47500.0	59100.0	59076.2	6123.2	48300.0	71300.0	54700.0	5275.5	45200.0	63400.0
Cd	0.0	0.0	0.0	0.0	0.0	0.0	0.0	0.0	0.0	0.0	0.0	0.0
Co	6.8	0.5	6.1	7.4	6.4	0.4	5.4	7.1	6.3	0.4	5.8	6.9
Cr	14.5	10.3	0.0	23.1	9.9	10.4	0.0	23.1	4.6	8.7	0.0	21.3
Cu	9.3	0.4	8.8	10.0	19.9	45.9	8.7	225.0	9.4	0.5	8.8	10.4
Fe	15355.6	911.8	13900.0	16900.0	14823.8	920.6	12400.0	16600.0	13811.1	799.2	12700.0	14900.0
K	1181.1	98.5	1070.0	1390.0	1187.8	128.8	914.0	1390.0	1102.2	57.9	1010.0	1170.0
Mg	21355.6	1621.5	17600.0	23300.0	20585.7	1456.4	18000.0	23100.0	20544.4	1665.4	18800.0	22900.0
Mn	578.8	157.0	382.0	980.0	533.4	46.8	432.0	620.0	594.1	199.8	404.0	1010.0
Mo	0.0	0.0	0.0	0.0	0.0	0.0	0.0	0.0	0.0	0.0	0.0	0.0
Na	217.9	11.8	203.0	238.0	219.1	13.8	193.0	241.0	195.4	9.7	185.0	217.0
Ni	11.4	0.6	10.5	12.5	10.7	1.2	0.0	15.8	10.4	0.6	9.8	11.5
P	712.2	50.6	609.0	764.0	689.7	37.8	608.0	754.0	595.3	29.9	553.0	660.0
Pb	0.0	0.0	0.0	0.0	0.6	2.6	0.0	12.2	0.0	0.0	0.0	0.0
S	797.1	60.8	679.0	912.0	885.3	71.1	734.0	1020.0	948.4	88.7	821.0	1090.0
Sb	0.0	0.0	0.0	0.0	0.0	0.0	0.0	0.0	0.0	0.0	0.0	0.0
Se	0.0	0.0	0.0	0.0	0.0	0.0	0.0	0.0	0.0	0.0	0.0	0.0
Sn	0.0	0.0	0.0	0.0	0.0	0.0	0.0	0.0	0.0	0.0	0.0	0.0
Sr	36.9	2.0	33.6	40.0	40.5	3.1	35.3	47.2	38.2	2.4	33.7	41.4
Ti	464.1	53.1	408.0	579.0	467.5	52.5	340.0	551.0	515.3	43.0	461.0	583.0
Tl	0.0	0.0	0.0	0.0	0.0	0.0	0.0	0.0	0.0	0.0	0.0	0.0
U	0.0	0.0	0.0	0.0	0.0	0.0	0.0	0.0	0.0	0.0	0.0	0.0
V	23.7	1.7	21.2	26.5	22.6	1.6	17.9	24.4	22.1	1.3	20.3	24.8
Zn	34.0	3.9	27.1	39.9	35.6	11.8	29.4	87.6	31.6	2.6	28.9	36.0
